# Reconstruction of hundreds of reference ancestral genomes across the eukaryotic kingdom

**DOI:** 10.1038/s41559-022-01956-z

**Published:** 2023-01-16

**Authors:** Matthieu Muffato, Alexandra Louis, Nga Thi Thuy Nguyen, Joseph Lucas, Camille Berthelot, Hugues Roest Crollius

**Affiliations:** 1 Institut de Biologie de l’École Normale Supérieure, Centre National de la Recherche Scientifique Unité Mixte de Recherche 8197, Institut National de la Santé et de la Recherche Médicale U1024, Université PSL, Paris, France; 2 Present Address: Wellcome Sanger Institute, Wellcome Genome Campus, Hinxton, UK; 3 Present Address: Institut Pasteur, Université Paris Cité, Centre National de la Recherche Scientifique Unité Mixte de Recherche 3525, Institut National de la Santé et de la Recherche Médicale UA12, Comparative Functional Genomics group, Paris, France

**Keywords:** Evolutionary genetics, Phylogenetics, Comparative genomics, Evolutionary biology, Phylogenomics

## Abstract

Ancestral sequence reconstruction is a fundamental aspect of molecular evolution studies and can trace small-scale sequence modifications through the evolution of genomes and species. In contrast, fine-grained reconstructions of ancestral genome organizations are still in their infancy, limiting our ability to draw comprehensive views of genome and karyotype evolution. Here we reconstruct the detailed gene contents and organizations of 624 ancestral vertebrate, plant, fungi, metazoan and protist genomes, 183 of which are near-complete chromosomal gene order reconstructions. Reconstructed ancestral genomes are similar to their descendants in terms of gene content as expected and agree precisely with reference cytogenetic and in silico reconstructions when available. By comparing successive ancestral genomes along the phylogenetic tree, we estimate the intra- and interchromosomal rearrangement history of all major vertebrate clades at high resolution. This freely available resource introduces the possibility to follow evolutionary processes at genomic scales in chronological order, across multiple clades and without relying on a single extant species as reference.

## Abstract (web summary)

Using an algorithm for ancestral genome reconstruction, the authors present 624 ancestral genomes for vertebrates, plants, fungi, metazoans and protists and reconstruct the chromosomal rearrangement history of all major vertebrate clades.

## Main

Biological sequences have long been recognized as a document of evolutionary history^1^, where accumulated mutations record relationships between species and the dynamics underlying their evolution. Given sufficient genetic information across species, the temporal accumulation of these mutations can be traced back in time to reconstruct sequences and genomes in their long-lost common ancestors. These ancestral reconstructions are the backbone of much of today’s methodologies in molecular evolution, including phylogenetic trees^2–4^ and sequence selection tests^5,6^. The reconstruction of ancestral sequences, and especially genes, has been extensively studied since the dawn of sequencing: mature methods exist to retrace the history of sequence substitutions and leverage changes in substitution dynamics to answer specific evolutionary questions. However, DNA mutations are not limited to sequence substitutions: genomes are also affected by larger scale mutational events such as duplications, deletions, sequence inversions or chromosomal rearrangements, all of which can affect genome function, species fitness and evolution. In extant species, large-scale mutations are a major determinant of disease because they can disrupt functional sequences^7–9^ and reorganize functional structures within the genome^10–12^. From an evolutionary viewpoint, large-scale mutations are a well-documented source of innovations: they can produce new genetic combinations that contribute phenotypic novelty^13,14^ but can also have more indirect effects such as locally suppressing recombination^15,16^, favouring allele hitchhiking and rapid selection^17,18^. For example, genomic rearrangements have been shown to associate with changes in brain gene expression between humans and chimpanzees^19^, to underlie the evolution of intersexual development in moles^20^ and variations in reproductive morphs in ruffs^21^. Despite their tremendous functional and evolutionary importance, large-scale mutational events are less extensively studied and not as well understood than sequence substitutions. In particular, the reconstruction of ancestral genomes and karyotypes lags behind that of ancestral sequences, making it difficult to study the evolutionary dynamics and impact of rearrangements, duplications and deletions over many species and within rigorous theoretical frameworks.

With the advent of massive sequencing projects ambitioning to obtain high-quality reference genomes for thousands of species across all kingdoms of life^22^, evolutionary genomics faces both fresh opportunities and serious challenges to integrate this flow of data into usable comparative frameworks. Along with whole-genome alignments^23^, ancestral genome and karyotype reconstructions across large clades is one of the most promising outcomes of these projects. The goal of these reconstructions is to provide a plausible organization of genomic sequences in one or many extinct common ancestors of a group of species of interest. Several palaeogenomic strategies have been explored to reconstruct the sequence content and ordering of ancestral genomes. Methods based on double-cut-and-join algorithms endeavour to reconstruct rearrangement scenarios resulting in observed extant genome structures^24,25^. These methodologies are increasingly computationally expensive and in many cases intractable for sets of large, complex genomes, which at this time have only been overcome by substantially reducing reconstruction resolution^26–28^. Other methods attempt to reconstruct a parsimonious sequence ordering in the ancestor based on orthologous sequence adjacencies in extant genomes, under the assumption that genomic rearrangements are unlikely to result in the same sequence organization several times independently. These methods can be applied to different types of markers, typically either alignable sequence blocks or individual genes, and are appropriate for small^29^ and large genomes such as vertebrates or plants^30,31^. However, it is unclear whether current methods can provide high-resolution reconstructions and scale to the large genomic resources available in comparative genomics databases. At this time, only two ancestral genomic reconstruction resources are widely available to the community: AncestralGenomes^32^, which provides 111 ancestral gene content reconstructions but not their order (‘bags of genes’), and DESCHRAMBLER^33^, which offers chromosome-complete reconstructions for 7 mammal and 14 bird ancestors but with limited subchromosomal resolution (100–300 kb sequence blocks) and dependent on a reference genome. In this study, we introduce a new resource containing 624 ancestral genomes reconstructed over the vertebrate, plant, fungi, metazoan and protist clades, at gene-scale resolution, where a third of the ancestral genomes reaches chromosomal-complete assemblies. This drastic change in magnitude is powered by an iterative, parsimony-based ancestral genome reconstruction algorithm, named AGORA (Algorithm for Gene Order Reconstruction in Ancestors), which we describe in this article. We show that AGORA is efficient, flexible and scales to integrate hundreds of large genomes, to reconstruct their common ancestors at every node in the species phylogeny with relatively modest computational costs. Along with the open-source algorithm, all precomputed ancestral genome reconstructions are publicly available in the Genomicus^34,35^ database (https://www.genomicus.bio.ens.psl.eu/genomicus) and benefit from the full browsing and comparative genomics tool infrastructure of the database. The database is regularly updated since 2010 to reflect reference genome improvements and represents a perennial resource for high-quality, high-resolution ancestral genomes for the molecular evolution community across disciplines and model phylogenetic clades.

## Results

### A resource of ancestral genomes for evolutionary genomics

**Fig. 1:**
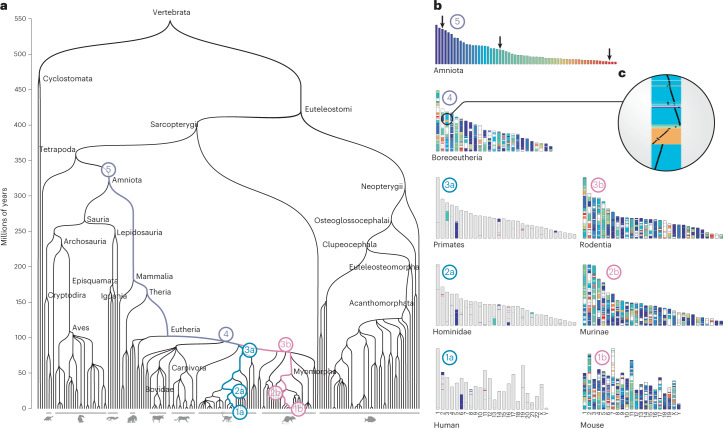
Reconstructing vertebrate ancestral genomes. **a**, Species phylogeny of vertebrates encompassing genomes stored in Ensembl v.92 with indications of the eight ancestral genomes detailed in **b** and the evolutionary path that they mark out. **b**,**c**, High-resolution ideograms of ancestral genome reconstructions (**b**) starting from the Amniota genome (5) and the descendant Boreoeutheria genome (4), where a region on the third chromosome is expanded to highlight the evolution of gene organization with respect to the Amniota genome (**c**). In the primate lineage (3a, 2a, 1a) only the evolution of the three Amniota chromosomes indicated by an arrow are depicted in colour, while in the Rodentia lineage (3b, 2b, 1b), the evolution of all Amniota chromosomes is shown.

To facilitate the investigation of chromosomal and local genome dynamics across evolution, we developed an extensive resource of ancestral genome reconstructions that spans large portions of the eukaryotic tree of life. This resource is based on an algorithm named AGORA, which computes highly contiguous, near-exhaustive reconstructions of the ancestral gene order at every bifurcation in the species tree, based on gene order information in the extant species of the clade (Fig. 1a). While AGORA can be installed as a standalone package for tailored research applications, we routinely precompute and release the complete set of ancestral vertebrate genomes for every update of the Ensembl database and for a broad selection of plant and fungi clades as part of the Genomicus synteny database^36^. At the time of submission, Genomicus contains a total of 624 ancestral genomes readily available for download across the vertebrates, plants, metazoa, protists and fungi databases (Supplementary Data 1). These ancestral genomes can be explored and manipulated using the different utilities of the Genomicus web server^36^ to perform karyotype comparisons, extraction and evolutionary tracing of conserved synteny blocks (Fig. 1b), and local gene–gene synteny visualization across ancestral and extant species (Fig. 1c). A partial draft version of AGORA, combined with extensive manual curation, has previously been used to reconstruct the Brassicacea^37^ and Amniota^38^ ancestors, illustrating several of these applications.

### AGORA is an algorithm to reconstruct ancestral gene order

**Fig. 2:**
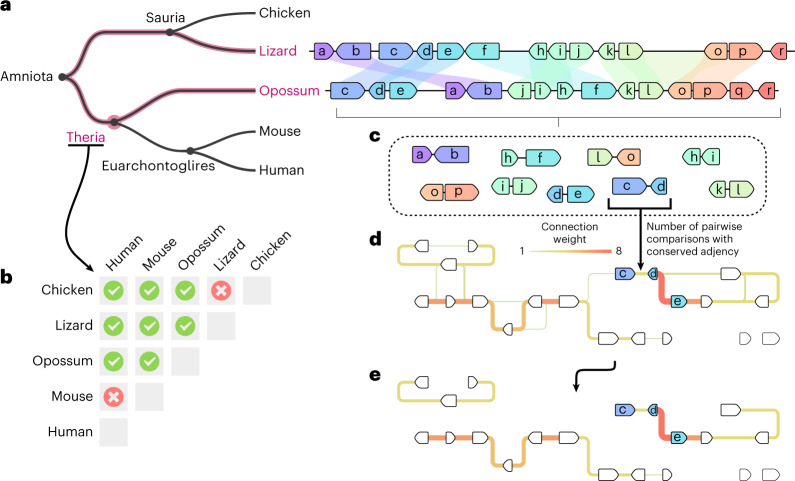
Principle of the AGORA approach. **a**, Conserved gene adjacencies are identified between all genome pairs that are informative for a given target ancestral genome. A portion of the lizard and opossum genomes are shown, with gene adjacencies joined by a pale coloured shape when conserved, thus supporting their prior occurrence in Theria. **b**, All comparisons between genomes that are joined in an evolutionary path intersecting the target ancestor are informative (green ticks) while comparisons between genomes that diverged after the target ancestor are uninformative (red crosses). **c**,**d**, Conserved adjacencies observed in each pairwise comparison (**c**) are collected in a graph structure (**d**) where nodes are genes and links are conserved adjacencies weighted by the number of times they have been observed in pairwise genome comparisons. **e**, The linearization of this graph by traversing the links of maximal weight provides contiguous and parsimonious ancestral gene order reconstructions.

AGORA is a parsimony-based algorithm that estimates the content and order of genes in the ancestor of a group of extant species for which reference genomes are available (Fig. 2 and Supplementary Fig. 1). Briefly, the method iteratively extracts commonalities between pairs of extant genomes to infer characteristics inherited from their last common ancestor and present in every ancestor along the evolutionary branches leading to each extant genome. AGORA takes as input a forest of gene phylogenetic trees, corresponding to all the gene families present in the extant genomes with their orthologous and paralogous relationships, and the gene orders in each extant genome. First, AGORA uses the phylogenies of extant genes to infer the gene content of every ancestor along the species tree (Supplementary Fig. 2). Second, AGORA compares the gene orders of every pair of extant species to identify orthologous genes adjacent and in the same orientation in both species and presumably inherited from their last common ancestor (Fig. 2a). For every ancestor in the species tree, the algorithm extracts the subset of informative pairwise extant species comparisons (Fig. 2b) and integrates the gene adjacency comparisons into a weighted graph, where nodes represent ancestral genes and edge adjacencies are supported by pairwise extant species comparisons. The weights correspond to the number of comparisons supporting that these genes were adjacent in this ancestor (Fig. 2c,d). Ideally, this process would result in a linear graph representing the ancestral gene order because genome rearrangements are unlikely to produce the same gene adjacencies independently in different lineages^39–42^. However, errors in the resolution of orthologues and paralogues in the original gene trees can result in branching in the graph. AGORA linearizes the graph by iteratively removing low-weight edges to obtain a parsimonious reconstruction of the oriented gene order in the ancestral genome (Fig. 2e). AGORA includes extensions of this algorithm to deal with larger errors in the input gene trees by identifying a set of constrained genes that are close to being single-copy in most species, and can be reliably used for gene order reconstruction. In this mode, AGORA adds the non-constrained genes in a second stage. The algorithm is presented in detail in the Supplementary Information (Supplementary Figs. 1–9). The in silico performance of AGORA has been tested on a previously used benchmark of genome evolution simulations^33^, achieving 98.9% agreement with the reference (sensitivity 99.3%, precision 99.6%; Methods), similar to other state-of-the-art ancestral genome reconstruction methods^33^. On a different, more realistic benchmark based on simulations that are not restricted to single-copy genes, AGORA achieves 95.4% agreement, while DESCHRAMBLER’s performance drops to 68.6% (Supplementary Information, ‘benchmarks against simulations’), highlighting AGORA’s ability to successfully deal with gene duplications and other complex evolutionary scenarios.

In practice, AGORA is highly flexible because it only requires the protein-coding gene annotations of the extant species and the set of precomputed gene trees in a standard format, which can be downloaded from a variety of genome resource initiatives for many species groups. For example, while the vertebrate ancestral genome reconstructions provided on the Genomicus server are all based on extant genomes annotated by Ensembl, plant and fungi ancestral genomes are based on genome annotations generated by a range of methods and laboratories worldwide. AGORA can be used with other markers than protein-coding genes, such as conserved non-coding elements; however, due to unreliability of phylogenetic trees for those sequences, we recommend limiting the reconstructions to the order of protein-coding genes for best performance. AGORA can also be used iteratively to assemble blocks of markers and scaffold them over several rounds of reconstruction into larger contiguous ancestral regions (CARs). We propose several workflows customized for different clades and applications as part of the AGORA package (Supplementary Fig. 1).

In this study, to demonstrate the capabilities of AGORA, we used two datasets from distant eukaryotic clades, with different numbers of species, genes and variable gene tree reliability: (1) a dataset of 93 vertebrates and 5 outgroups and their 23,528 gene trees, including a total of 1,814,614 extant protein-coding genes, and leading to the reconstruction of 81 ancestral genomes; and (2) a dataset of 58 plant genomes and 8 outgroups, corresponding to 48 ancestral genomes (Methods, Supplementary Data 4 and Supplementary File 1).

### Reconstruction of key chromosome-scale ancestral genomes

**Fig. 3:**
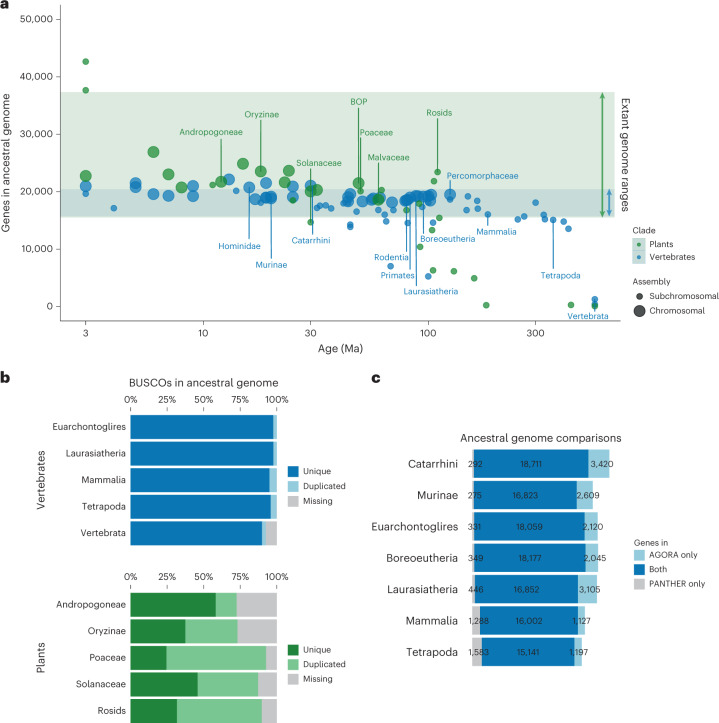
Completion of ancestral genomes reconstructed by AGORA. **a**, Gene content and assembly continuity of 77 vertebrate and 33 plant ancestral genomes reconstructed by AGORA. The ranges of gene contents of extant vertebrate and plant genomes are highlighted in blue and green shading, respectively. Very young (<2 Ma) or very old (>550 Ma) ancestors are not represented. Chromosomal and subchromosomal assemblies are as defined in the Methods. The BOP ancestor stands for the ancestor of the Bambusoideae, Oryzoideae and Pooideae groups. **b**, Representation of BUSCO genes in AGORA’s ancestral genomes. Plant genomes, which have undergone rounds of WGDs, frequently contain a large fraction of duplicated genes. **c**, Comparison of ancestral gene contents inferred by reconstructions from AGORA and PANTHER.

For every ancestral genome, we provide two valuable results: the gene set and an assembly of their ancestral organization. To evaluate the completeness and accuracy of the ancestral gene sets, we first compared the total number of genes inferred in an ancestor to those of its descendant extant genomes. While very distant genomes can contain widely different numbers of genes, AGORA is designed to be used within clades where synteny is reasonably conserved, such as vertebrates, grasses or *Saccharomycetales* yeasts, and where genomes typically contain similar numbers of genes. We found that our methodology accurately estimated ancestral gene contents that were consistent with those of the descending clades, up to evolutionary distances of over 300 million years ago (Ma) (Fig. 3a). We also find that the vast majority of clade-relevant benchmark universal single-copy orthologue (BUSCO)^43^ reference sets are present as single-copy genes in our inferred ancestral gene sets (Fig. 3b). In addition, we also confronted our inferred ancestral gene contents for seven key vertebrate ancestors to those calculated by Ancestral Genomes, another effort to estimate the ancestral gene content, but not gene order, at different evolutionary nodes^29^. Ancestral Genomes relies on the PANTHER database^44^ and therefore uses an independent set of extant genomes and gene trees. AGORA and Ancestral Genomes both inferred highly similar gene contents for the same ancestors (Fig. 3c).

The other output of AGORA is the reconstruction of the putative gene order in each ancestral genome along the species tree. The quality of an ancestral genome reconstruction can be evaluated by two criteria, that is, contiguity and consistency with evolutionary and biological evidence. Contiguity represents the size of the genomic regions that can be assembled into CARs, akin to measures of assembly quality for reference genome sequences. For 37 vertebrate ancestral genomes and 13 plant ancestral genomes in our test set, we obtained chromosome-scale assemblies with a small number of long CARs containing hundreds of ordered and oriented genes, corresponding to a best approximation of the ancestral karyotype (Fig. 3a). These chromosome-level assemblies include over 70% of the ancestral genes, which is comparable to well-assembled extant reference genomes in those clades (Supplementary Fig. 10). Most other ancestral genomes are assembled into fewer than 100 subchromosomal gene blocks containing over 70% of the ancestral gene content (Supplementary Fig. 11).

As expected, the contiguity of ancestral genome reconstructions was high overall in recent ancestors and decreased sharply after 100 Ma, decaying to large numbers of short, unassembled gene blocks for very ancient ancestors such as the Tetrapoda and Vertebrata ancestors (Fig. 3a). However, perhaps counterintuitively, AGORA performs better in some key older ancestors than in comparatively younger ancestral genomes. For example, the genome of Boreoeutheria, the ancestor of most placental mammals (approximately 95 Ma), is a near-complete assembly consisting of 25 large CARs covering 18,430 genes (80% of the total ancestral genome), while the genome of Afrotheria, the ancestor of the elephant and hyrax (approximately 90 Ma), is appreciably less contiguous with 70% of genes in 83 CARs. This reflects the position of these ancestors in the species tree relative to the sampling of sequenced extant genomes. As demonstrated previously^45^, ancestors that precede evolutionary radiations are ideally positioned for ancestral genome reconstruction because their many outgroup and descendant lineages offer a large number of informative pairwise comparisons (*N*_i_). Overall, AGORA’s ancestral reconstruction contiguity correlates with the *N*_i_/age ratio (Supplementary Fig. 12). Because sequencing efforts have largely targeted organisms within species-rich phyla, such as placental mammals or monocotyledon plants, the key ancestors to these widely studied subclades are particularly well reconstructed by our methodology, which should be of high value to evolutionary and functional studies. Ultimately, however, with the advent of massive sequencing undertakings such as the Vertebrate Genome Project, genome documentation in undersampled clades will increase dramatically and we expect that most ancestral genomes in the Genomicus database will eventually become chromosome-level assemblies.

### Support from cytological evidence and in silico palaeogenomes

**Fig. 4:**
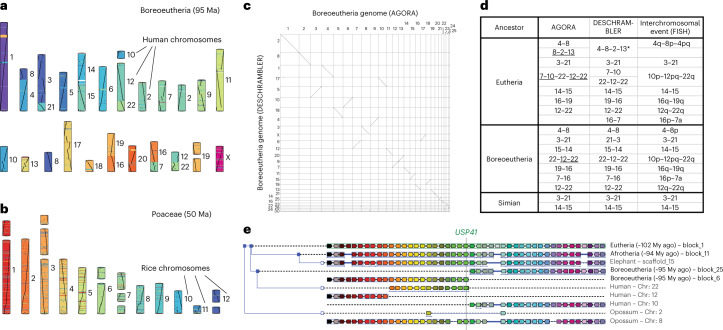
AGORA ancestral genome reconstructions compared to extant genomes and state-of-the-art ancestral reconstructions. **a**, The Boreoeutheria karyotype inferred by AGORA (the 25 largest CARs), coloured according to gene locations on human chromosomes, as indicated to the right of each CAR. **b**, The Poaceae karyotype inferred by AGORA (the 19 largest CARs), coloured according to gene locations on *Oryza sativa* chromosomes, as indicated to the right of each CAR. **c**, Collinearity of the Boreoeutheria ancestral genome reconstructed by AGORA with the genome reconstructed by DESCHRAMBLER^33^. **d**, Comparisons of computational reconstructions by AGORA and DESCHRAMBLER and Zoo-fluorescence in situ hybridization (FISH) linkage groups inferred for three key mammalian ancestors. Human chromosomes in ancestral linkage are indicated with hyphens. The Eutheria bolded linkage group 10–22–12 is documented in more detail in **e**. The underlined linkage groups are documented in Supplementary Fig. 14. DESCHRAMBLER reconstructed a linkage group between parts of human chromosomes 4, 8, 12 and 3 (asterisk) in disagreement with FISH evidence and AGORA when used on Ensembl v.92 data; however, this linkage group is also reconstructed by AGORA on Ensembl data v.102, suggesting an ambiguous ancestral linkage state (Supplementary Fig. 15). **e**, Gene adjacencies around the *USP41* gene in extant species support the linkage of fragments of human chromosomes 10, 22 and 12 in the Eutheria ancestor. Orthologous genes are shown as arrows in matching colours, pointing in the direction of transcription. Opossum and elephant have both retained the ancestral organization at this locus, which has been rearranged in the human genome.

The accuracy of ancestral genome reconstructions is appreciably more difficult to evaluate than completion because the true ancestral genome sequences are inaccessible at the evolutionary scales we study. However, several ancestral genomes have garnered longstanding interest from the evolutionary genomics community, resulting in a large body of biological evidence regarding their overall organization. In vertebrates, one of the most studied ancestral genomes is Boreoeutheria, the 95 million-year-old ancestor to most placental mammals including primates, rodents, hooved mammals and carnivores, with the exception of afrotherians (elephants) and xenarthrans (tree sloths, anteaters, armadillos), along with the Eutheria ancestor (102 million-year-old, ancestral to boreoeutherian mammals and afrotherians) and the Simian ancestor (45 million-year-old, ancestral to platyrrhine and catarrhine primates). Landmark ancestral Eutheria, Boreoeutheria and Simian karyotypes have previously been reconstructed by integrating dozens of mammalian homology comparisons using fluorescent DNA probes, a technique known as chromosome painting^46,47^. This analysis suggested that the ancestral placental genome consisted of 23 pairs of chromosomes and traced the large-scale rearrangements that resulted into the karyotypic arrangement of the human genome. The Boreoeutheria ancestral genome organization inferred by AGORA contains 25 large CARs and is highly congruent with the cytogenetically based reference karyotype (Fig. 4a). AGORA recovers all ancestral chromosomal arrangements supported by cytogenetic evidence without requiring manual assembly or curation. The only exception is the ancestral linkage of human chromosomes 10 and 12 alleged by cytogenetic data (Fig. 4c–e), which is supported neither by AGORA nor by the state-of-the-art reconstruction by DESCHRAMBLER or other in silico ancestral genome reconstruction methods^33^. Detailed manual investigation of inconsistencies between the ancestral reconstructions by AGORA and the cytogenetic references revealed that most differences are the result of the lower resolution of the chromosomal painting methodology and confirmed our proposed assembly (Supplementary Figs. 14 and 15). At the infrachromosomal scale, we found that the genomic organization of the Boreoeutheria genome inferred by AGORA is in near-perfect agreement with that of DESCHRAMBLER (Fig. 4c, Supplementary Fig. 16 and Methods). However, our reconstructed Boreoeutheria genome is more complete and includes the ancestral locations of an additional 2,023 genes (8% of the ancestral gene set) due to operating at a higher resolution. AGORA also fared better by including more species and more recent assemblies than DESCHRAMBLER. Altogether, these results support that the gene-based reconstruction algorithm of AGORA is highly consistent with current ancestral reconstruction methods, while providing a notable increase in resolution for the study of local genomic events. We further tested the robustness of AGORA to varying input datasets by reconstructing an alternative Boreoeutheria ancestral genome using gene families from hierarchical orthology groups built with OMA^48^, a completely different gene orthology inference pipeline from Ensembl Compara. Both reconstructions were remarkably convergent with over 96% similarity (Supplementary Information, ‘Comparison between Ensembl Compara and OMA hierarchical orthology groups’ and Supplementary Fig. 17), supporting that AGORA performs well regardless of gene orthology data sources.

Finally, we also examined the genome of Poaceae, the 50 million-year-old ancestor of grasses, reconstructed by AGORA to an earlier reference ancestral karyotype^49^ obtained by another parsimony-based method to reconstruct ancestral adjacencies^30^. Again, the ancestral genome reconstructed by AGORA closely recapitulates the state-of-the-art knowledge regarding the organization of the ancestral grass karyotype (Fig. 4b), while providing access to a fine-scale reconstruction of the ancestral gene order.

### A scalable framework to integrate genomes across phylogenies

A major strength of AGORA resides in its ability to compute the gene order of every ancestor in a phylogeny using different subsets of the same extant genome comparisons. In a context where new species genomes are being sequenced with increasing speed and accuracy, comparative genomics need methods that can integrate evolutionary information along the species tree and across lineages without relying on a single extant genome as reference. Using the legacy architecture of the Genomicus synteny database^34,35^, which is updated with every new release of the Ensembl database, we tested how our methodology scales with the number of extant reference genomes available as well as their quality (Supplementary Fig. 13). Ensembl Compara v.101 included the reference sequences of 264 vertebrate species and five outgroups, for a total of 5,539,325 extant protein-coding genes organized into 62,478 gene trees. Using this information, AGORA reconstructed a total of 265 ancestral genomes along the species tree in 6 h and 50 min on a Linux machine with four central processing units and approximately 80 GB of random access memory (Supplementary Data 2). Therefore, AGORA is computationally inexpensive and can be run on a desktop machine for small-to-medium datasets. However, AGORA can also be parallelized and is optimized for usage on a computing cluster for large applications and database updates.

Overall, the quality of key ancestral genomes increases as new extant genomes are included in the database (Supplementary Fig. 13). The introduction of high-quality reference genomes in under-represented clades over time has contributed to the reconstruction of previously inaccessible ancestors, such as Strepsirrhini, the ancestor of lemurs, bushbabies and lorises, and more recently Chiroptera, the ancestor of bats. Interestingly, we observed that even the inclusion of low-contiguity, fragmented genomes markedly improves ancestral genome reconstructions. For instance, including low-contiguity genomes more than doubles the median value (G50; Methods) for the reconstructed Amniota genome (Supplementary Information, ‘Impact of low-contiguity assemblies’). This is likely because different reference genomes are generally assembled independently and assembly errors rarely produce the same erroneous gene arrangements from one genome to the next. Because AGORA only considers conserved gene adjacencies as potentially ancestral, the additional information from correctly assembled scaffolds offsets the noise introduced by assembly errors, which are discarded as not conserved. Therefore, we argue that the inclusion of low-cost, fragmented reference genomes in comparative genomics databases serves a purpose beyond gene-based analyses.

### Ancestral genomes as backbones for evolutionary studies

**Fig. 5:**
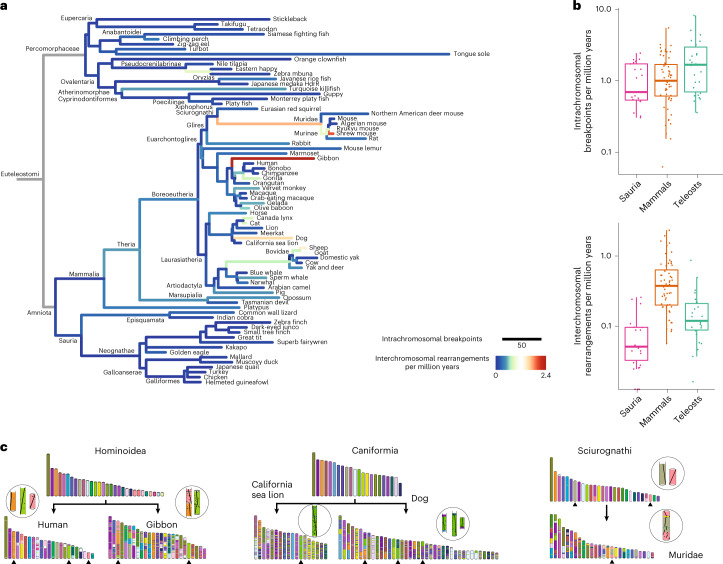
Vertebrate genome evolutionary dynamics. **a**, Phylogenetic tree of 74 extant and 73 ancestral genomes, where branch lengths represent the number of breakpoints computed between successive nodes. The colours represent the rate of interchromosomal rearrangements. The branches in grey connect ancestral genomes to their Euteleostomi root, which is too fragmented as a genome reconstruction to serve as reference for computing breakpoints and rearrangements. **b**, Distributions of breakpoints and rearrangement rates represented in **a**, broken down into three taxonomic groups: saurians (birds and reptiles); mammals; and teleosts (fish). The centre line of each plot corresponds to the median, the box edges correspond to the first and third quartiles, the whiskers correspond to 1.5× the interquartile range. **c**, Examples of rearrangements in lineages with notable rates of evolution. The black upward arrowheads point to chromosomes that are shown enlarged in the circles, with individual orthologous genes drawn as black dots.

In this section, we experimented the paradigm shift. This consisted of studying genome evolution from the perspective of multiple reconstructed ancestral genomes. We first revisited known observations generated by traditional comparative genomics based on extant genomes. As a case study, we used ancestral reconstructions to investigate the patterns of karyotypic rearrangements that occurred during the evolution of mammals, birds and ray-finned fish (Fig. 5). These three groups represent the main jawed vertebrate (Euteleostomi) lineages, whose respective chromosomal dynamics have been documented using comparative genetics, cytogenetics and genomics approaches across different taxonomic groups. We selected 73 well-reconstructed ancestors and their 74 extant descendants (15 birds and reptiles, 41 mammals, 18 fish; Methods) from the Genomicus Vertebrates database v.102, which contains a total of 269 extant and 265 ancestral genomes. We then compared consecutive genomes on all 131 branches of the phylogenetic tree, representing a combined time of about 5 billion years of independent evolution, and traced gene adjacencies that were rearranged on each branch (Methods). In total, we identified 5,749 rearrangement breakpoints that occurred along the 131 branches (average rate 1.17 breakpoint per million years), most of which are intrachromosomal. We also identified 1,370 interchromosomal rearrangements (translocations, fusions or fissions) with an average rate of 0.28 rearrangement per million year (Fig. 5a and Supplementary Data 3). These rearrangement rates are lower bound values because rearrangements occurring between genes without disrupting gene order or orientation cannot be observed (Discussion). Comparing rates per million years, and restricting the analysis to the 105 branches longer than 5 Ma to avoid small sample distortions, we confirm that birds and reptiles have more stable chromosomal structures than mammals, as reported previously^50,51^, with lower rates of interchromosomal rearrangements (*P* = 3.8 × 10^−6^, Wilcoxon rank-sum test; Fig. 5b). Fish in turn display higher intrachromosomal breakpoint rates than mammals, birds and reptiles (teleosts versus saurians, *P* = 0.0181; teleosts versus mammals, *P* = 0.0532, Wilcoxon rank-sum test), which is consistent with the rediploidization process following the whole-genome duplication (WGD) that occurred in this phylum^52^, yet they display a uniformly high karyotypic stability.

Interestingly, a few branches in placental mammals stand out as having strikingly high rearrangement rates. For instance, the gibbon lineage is the outlier of our analysis, having experienced 60 interchromosomal rearrangements in 25 My, confirming previous observations that this is a fast-evolving lineage compared, for example, to the human lineage^53^ (Fig. 5c). The dog genome was also subject to high rates of rearrangement, especially compared to its sister branch leading to the slowly evolving sea lion genome, which only changed through three chromosome fusions compared to their Caniformia ancestor^54^. The lineage leading to the Muridae is notable for a high rate of intrachromosomal breakpoints combined with multiple interchromosomal rearrangements but associated to a stable chromosome number, which is consistent with cytogenetic studies of different murid clades^55,56^. These examples revisit lineages that are known to be subject to fast evolutionary rates, underlining how AGORA reconstructions agree with current knowledge and represent a sound basis to explore and understand genome evolution.

**Fig. 6:**
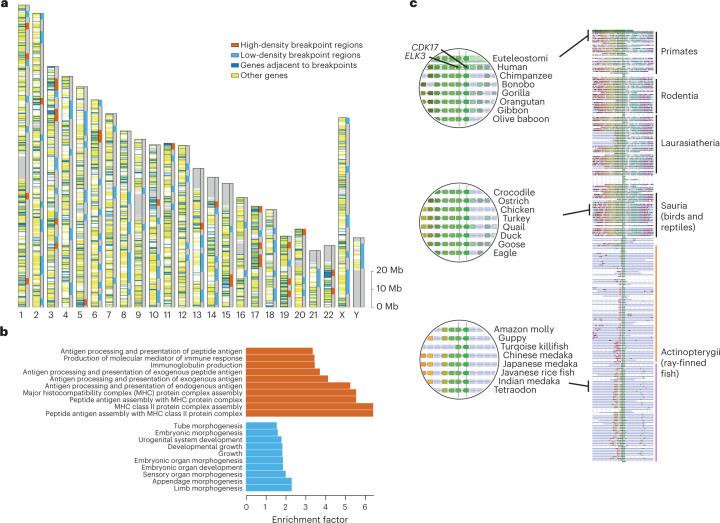
Breakpoint map and functional associations. **a**, Human chromosome map showing the 1,985 genes that flanked a breakpoint at least once during the evolution of Boreoeutheria (dark blue) and the genes that were never adjacent to a breakpoint (yellow). The genome was divided in 5 Mb non-overlapping windows and the red boxes show the top 28 windows (5%) richest in breakpoints, while the light blue boxes indicate the 123 boxes without any breakpoints. **b**, A GO enrichment test showed that breakpoint-rich and breakpoint-poor windows are enriched in genes with very contrasted GO biological functions. The 10 most enriched terms with an FDR < 5.10^−2^ and associated with fewer than 1,500 human genes in the complete genome for each gene category are shown. **c**, The *ELK3-CDK17* genes represent the most conserved gene adjacency in bony vertebrates (Euteleostomi). Right, The *EDK3-CDK17* locus is shown in a Genomicus v.106 phyloview, at the centre of a 45-gene window in the 187 extant Euteleostomi genomes where it is conserved. Genes and loci are not drawn to scale and each gene is represented by an arrow of fixed size pointing in the direction of transcription. The orange and purple vertical lines outline the two groups of teleost fish loci separated by the 3R WGD. Left, The three circles show a zoomed-in region with the *EDK3-CDK17* gene pair in head-to-head orientation, aligned over the vertical black line in each genome.

A key feature of AGORA reconstructions is that they are independently derived for each ancestor, enabling the investigation of evolutionary events in internal branches, between successive ancestral genomes (Fig. 5c, Sciurognathi to Muridae). We exploited this feature to investigate whether rearrangement breakpoints accumulate in specific genomic regions in mammals, where they may present an evolutionary advantage by providing new gene combinations. We collected 2,466 rearrangement breakpoints that occurred across all the boreoeutherian mammal lineages shown in Fig. 5a. This ‘breakpoint map’ recapitulates almost 1.4 cumulated billion years of genome reorganization, projected on the human genome as a reference. In total, 1,985 human genes are flanked by at least 1 breakpoint (Fig. 6a) and high and low breakpoint density regions are evident. To characterize these further, we identified the 5 Mb windows in the human genome with the highest density of breakpoints (top 5%) and those without breakpoints. A Gene Ontology (GO) analysis showed that high breakpoint intensity occurs near genes involved in the acquired immune system, while breakpoints are depleted in regions flanking genes involved in embryonic development (Fig. 6b), confirming on a broad scale previous observations^57,58^. Genomic regions involved in immunity are fast-evolving at the sequence level, typically interpreted as evidence of positive selection: in this study, we show that these regions are also fast-rearranging; further investigation may reveal whether genomic reorganization acts in concert with sequence evolution to produce functional novelty in these regions.

Finally, we took advantage of the unique standpoint provided by ancestral genomes to investigate which gene-to-gene interval is the most conserved in all bony vertebrates. Scanning the Euteleostomi ancestral genome, we selected the gene adjacency with the strongest support from 8,173 pairwise genome comparisons used to reconstruct this genome. The adjacency between the *ELK3* and *CDK17* genes is ancestral to bony vertebrates and remains conserved in 187 out of 192 descendant genomes available in the Ensembl 106 database (Fig. 6c). Interestingly, *ELK3* and *CDK17* coexpress sense–antisense messenger RNA transcripts in mouse neuronal cells^59^. Additionally, *ELK3* introns contain enhancer sequences that putatively target the *CDK17* promoter^60^. Potential complex regulatory functions may be associated with this locus because a sense–antisense transcript produced in the same cells can lead to double-stranded RNA, and in this case, also overlap the *ELK3* coding exon. Further investigation should reveal whether the same coexpression occurs in all Euteleostomi, which would suggest an ancestral function established early in vertebrate evolution and a possible explanation for the extensive linkage conservation at this locus.

## Discussion

Biology is a historical science but this historical dimension is often ignored because the records required to document ancestral states are missing. Without this chronological perspective, the reasons why contemporary biological systems are organized as they are will continue to elude us. In practice, this information gap hinders our ability to integrate conclusions across different living models and to draw the full benefits of comparative genomics. Ancestral genomes are fundamental blocks of the conceptual framework aiming to address this problem. They complement fossils as biological time points because they are a theoretical representation of the precise divergence between two lineages, while fossils represent true extinct species but whose exact phylogenetic position is often unclear. Because ancestral genomes encapsulate all the genes present in the ancestral organism and their structural organization, they will enable detailed investigations of developmental and metabolic pathways evolution, such as the expansion and contraction of specific gene families over time; the contribution of genome structure changes to evolutionary transitions and speciations; and the tracing of evolutionary innovations through reorganization of functional gene arrangements. Additionally, ancestral genomes can act as unique reference points to compare multiple descendant genomes, removing the bias of relying on an extant genome as central reference. This property makes them powerful tools to identify, measure and study lineage-specific genomic events and clade-wide trends.

Reconstructions of ancestral genomes by AGORA have a number of limitations. First, the method relies on the assumption of parsimony, which is widely used for both cytogenetic and marker-based bioinformatics reconstructions. This premise is reasonable because intergenic breakpoints are rare (fewer than ten per million years in eukaryotes) and conservative scenarios involving the fewest steps are likely to be correct in the vast majority of cases. However, breakpoint reuse can occur^61^ and will violate this assumption, which may result in non-reconstructed gene adjacencies (false negatives) in the AGORA reconstructions but will not create erroneous adjacencies (false positives). Thus, breakpoint reuse may cause reconstructions to be more fragmented but should not induce incorrect links between markers. It will, however, cause an underestimation of breakpoint rates as presented in Fig. 5, although there is no evidence that it should distort the relative rates between taxonomic groups. Conversely, a false positive adjacency present in a given ancestor but absent in the previous one and in the next one in a chronology, will give a false signal of breakpoint reuse. Other limits are less due to the method but are inherent to the underlying data. For example, in this study we used gene trees to define the set of ancestral genes to be ordered into chromosomes and to locate the set of descendant genes in extant genomes (orthogroups). Although we showed that two different sources of orthogroups (Ensembl and OMA) generate essentially the same Boreoeutheria genome, this may be different for more ancient genomes or poorer-quality gene trees. In particular, incorrect placement of duplication events will affect the number of ancestral genes and incorrect partitioning of extant copies under their ancestral duplicate copy will affect the adjacencies that can be deduced. This issue is amplified after WGD events, where all genes are duplicated at once, but can be mitigated by tree edition steps as implemented in SCORPiOs^62^. WGD are not obstacles per se for genome reconstruction. Several instances occurred in vertebrate, plant and fungi genome evolution and AGORA can reconstruct ancestral genomes at speciation nodes immediately flanking the event. This is the case, for example, between the Protacanthopterygii and Salmoninae in fish that flank a single WGD, and between the Malvids and Brassicaceae ancestors in plants that flank two successive WGDs. In each case, the classical ‘double-conserved synteny’ pattern^52,63,64^ is clearly visible across ancestral chromosome segments hundreds of genes long (Supplementary Fig. 18). The density of markers (that is, protein-coding genes) also limits the resolution of the reconstructions because intermarker space consists of blind spots where inversions contained within cannot be observed. As algorithms mature, ancestral genomes such as those presented in this study could become enriched with many more features, including non-coding sequences such as ancestral repeat elements, non-coding RNA genes or regulatory elements, and serve as organizational maps for reconstructed^65^ or fossil nucleotide sequences. Reaching this goal could alleviate some limitations of gene-based ancestral reconstructions by providing a much-increased resolution.

Because genome sequencing costs continue to decrease, reference genomes are becoming widely available for model and non-model species alike. At the time of writing, the NCBI database accounts for a total of 8,505 eukaryote, 32,172 bacterial and 1,909 archaeal whole-genome sequencing projects and dedicated efforts such as the Vertebrate Genome Project^66^ promise to deliver extensive phylogenetic coverage across many clades. Integrating sequence and genome organization evolution over such massive phylogenetic samplings remains a challenge. Many phylogenomics projects still rely on sequence alignments as a means to study how genome organization evolves^33,51^. Aligning whole genomes is computationally expensive, and while new methodologies are emerging to step up to the challenge^23,67^, the requirements to handle hundreds of genomes remain out of reasonable reach for many. Additionally, identifying conserved and rearranged regions from whole-genome alignments becomes technically difficult as phylogenetic distance increases, especially in large genomes where an important fraction of the sequence is non-coding and repetitive. Due to these limitations, the evolution of genome organization is typically studied at large scale, but low resolution, and/or in a limited sampling of species, often those included in publicly available, reference multispecies alignments. Marker-based ancestral genome reconstructions provide an alternative to methods based on whole-genome alignments by relying on gene phylogenies instead, which require much more modest computational infrastructures and scale up to hundreds of genomes with relative ease. In the future, as polymorphism information becomes available for more extant species, we may expect to see ancestral genomes move on from unique references to compendiums, representing structural genomic variation present at any given point in time and opening the door from increasingly sophisticated population genomics models of molecular evolution.

## Methods

### Data collection

Genes and gene trees were downloaded from Ensembl v.92 (ref. 68) and Ensembl Plants v.41 (ref. 69). Ensembl v.92 gene trees were edited for poorly supported duplication nodes as described previously^70^, as part of the standard build procedure for the Genomicus synteny database. Of note, this step only marginally improves ancestral genome reconstructions and is not a prerequisite to use AGORA. The species trees for the extant and ancestral genomes from Ensembl v.92 and Ensembl Plants v.41 are described in Supplementary File 1.

### Ancestral genome reconstructions

Ancestral gene sets and gene orders were reconstructed for 82 ancestors on Ensembl v.92 data using AGORA with 2 passes and a tree parameter of 0.35, and for 41 plant ancestors in 2 multi-integration passes without tree selection (Supplementary Data 4). The details of the AGORA algorithm, validations by evolutionary simulations, suggested procedure to select an optimal tree parameter and advances compared to earlier publications are detailed in the Supplementary Information (‘AGORA method’).

### Statistics on ancestral genomes

Ancestral genome contiguity was measured using the L70 and G50 metrics. L70 is the smallest number of CARs adding up to 70% of the total genome length, measured in gene units. G50 is the length of the ancestral CAR such that 50% of the total genome length, measured in gene units, is contained in larger CARs. Vertebrate chromosomal assemblies have an L70 < 100 and G50 > 450 and plant chromosomal assemblies have an L70 < 20 and a G50 > 450. These values correspond to well-assembled extant genomes (Supplementary Fig. 10) from these respective clades. Other assemblies were considered subchromosomal.

### Comparisons to reference ancestral gene sets

We downloaded the BUSCO sets v.3 (ref. 43) based on OrthoDB v.9 (ref. 71). BUSCO gene identifiers were converted to Ensembl gene IDs using the conversion tables provided by the OrthoDB. A BUSCO orthogroup is a set of near 1-to-1 orthologous genes across sequenced genomes of a relevant phylum. An ancestral gene inferred by AGORA was identified as a BUSCO if two or more of its extant descendant genes were contained in the same orthogroup. When a single ancestral gene had descendants in more than one BUSCO orthogroup, we chose the orthogroup with the highest overlap. We then computed the number of BUSCOs matched to a single ancestral gene, to two or more ancestral genes (dubious duplication) and absent from the ancestral genome reconstructed by AGORA (missing gene). Independent ancestral gene sets were downloaded from Ancestral Genomes^32^, based on PANTHER v.13.1 (ref. 44). Because Ancestral Genomes and AGORA use different sets of extant species, we only considered ancestral genes with descendants in one of their common species for comparison (human for all ancestors except Murinae and Laurasiatheria where mouse and dog were used, respectively). Ancestral Genomes ancestral genes were converted from UniProt knowledge base IDs to Ensembl gene IDs using the correspondence tables provided by Ensembl BioMart and compared with the gene sets in the ancestral genomes reconstructed by AGORA.

### Comparison between AGORA and DESCHRAMBLER eutherian ancestor

We compared AGORA’s v.92 eutherian reconstructions to DESCHRAMBLER’s^33^ (300 kb resolution: APCF_hg19_merged.map from http://bioinfo.konkuk.ac.kr/DESCHRAMBLER/). Because DESCHRAMBLER uses segments of the human genome as units of the reconstruction and was based on the hg19 genome assembly, we converted those regions to their protein-coding gene content and selected the genes still found in Ensembl v.92 and descendants of ancestral boreoeutherian genes. The Oxford grid plot was generated with the AGORA src/misc.compareGenomes.py script in ‘matrix’ mode.

### Vertebrate evolutionary dynamics

Ancestral genomes reconstructed by AGORA from Ensembl v.102 were filtered to retain the most contiguous reconstructions, resulting in 73 ancestral genomes with G50 > 230 and L70 < 40. Conserved syntenic blocks between successive ancestral genomes in internal branches, and between ancestral genomes and their extant descendant in terminal branches, were computed with PhylDiag^72^. Ends of blocks corresponding to likely evolutionary breakpoints were identified using ad hoc scripts. Orthologous genes between successive genomes were also compared in terms of their assignation to scaffolds or chromosomes larger than 200 genes using AGORA’s src/misc.compareGenomes.py script in ‘printOrthologousChrom’ mode. Groups of at least 20 genes relocating to more than 1 chromosome in a descendant genome, and inversely groups of at least 20 genes from 2 or more ancestral chromosomes relocating on the same descendant chromosome, were considered interchromosomal rearrangements. Breakpoint and rearrangement rates per million years were computed using branch length estimates from TimeTree^73^. A full description of the parameters and selection thresholds are provided in the Supplementary Information (‘Vertebrate genome evolutionary dynamics’).

### GO analysis

Human genes from Ensembl 106 contained in 5 Mb windows with the 5% highest number of breakpoints or with no breakpoints were tested for GO term enrichments (biological function) against the rest of the human genes, using the PANTHER web server^44^ (version 17.0). Enrichment was tested with Fisher’s exact test; terms with a false discovery rate (FDR) < 0.05 were retained. Control experiments with random selections of windows with the same gene densities as found in the 0-breakpoint windows and 5% richest windows did not show significant enrichment.

### Reporting summary

Further information on research design is available in the Nature Portfolio Reporting Summary linked to this article.

## Supplementary information

Supplementary Information Supplementary Methods, Results, Tables 1–3 and Figs. 1–18.

Reporting Summary

Supplementary Data 1 Statistics about the 629 extant genomes used in this study.

Supplementary Data 2 Rearrangement rates per branch in vertebrates.

Supplementary Data 3 Benchmark of computation times of the AGORA algorithm.

Supplementary Data 4 Statistics on ancestral genome reconstructions in vertebrates and plants.

## Data Availability

Ancestral genomes have been precomputed for approximately 200 vertebrate (depending on the release), 41 plant and 222 fungi genomes and are available on the Genomicus database FTP server (ftp://ftp.bio.ens.psl.eu/pub/dyogen/genomicus/). These ancestral genomes can also be explored visually within the Genomicus^35^ synteny browser (http://www.genomicus.bio.ens.psl.eu/genomicus). Ancestral genomes and the data used in this article for analysis are archived on a *Zenodo* repository (10.5281/zenodo.7479507)^74^.

## Code availability

The source code of AGORA, user instructions and a test dataset are available for download from https://github.com/DyogenIBENS/Agora.
